# S100A9 enhances tumor immune suppression and cancer cell survival in small cell lung cancer

**DOI:** 10.1038/s41419-025-08102-0

**Published:** 2025-10-31

**Authors:** Manish Charan, Sanjay Mishra, Konstantin Shilo, Xiaoli Zhang, Ajeet K. Verma, Pratyusha Ghanta, Amy Webb, Ramesh K. Ganju

**Affiliations:** 1 Department of Pathology, College of Medicine, The Ohio State University, Columbus, OH USA; 2 Comprehensive Cancer Center, The Ohio State University, Columbus, OH USA; 3 Center for Biostatistics, The Ohio State University, Columbus, OH USA; 4 Department of Biomedical Informatics, The Ohio State University, Columbus, OH USA

**Keywords:** Cancer microenvironment, Small-cell lung cancer

## Abstract

Small cell lung cancer (SCLC) is a highly aggressive form of lung cancer associated with a poor prognosis. However, there have been few advancements in improving the survival of SCLC patients in recent decades. This study shows that the S100A9 protein is highly expressed in SCLC patients and several highly aggressive SCLC cell lines. Furthermore, we showed that S100A9 expression inversely correlates with overall survival in SCLC patients. S100A9 increases the survival and migration of SCLC cells by activating Akt and GSK3α/β/Snail pathways. In addition, S100A9 reduces tumor cell autophagy through MAGE-A3. S100A9 depletion or pharmacological inhibition using tasquinimod reduced tumor growth and metastasis in vivo. Importantly, we observed that S100A9 downregulation or tasquinimod treatment alone or combined with cisplatin reduces the recruitment of MDSCs. Furthermore, tasquinimod treatment alone or combined with cisplatin significantly enhanced tumor infiltration of activated CD8+ (CD69-positive) T cells. Overall, our results, for the first time, show that S100A9 enhances SCLC progression and metastasis by altering the tumor microenvironment, and its inhibition using tasquinimod alone or in combination with chemotherapy could be developed as a promising therapeutic strategy for SCLC.

## Introduction

Small cell lung cancer (SCLC) is a highly aggressive lung malignancy, accounting for ~15% of all lung cancer cases [1]. The aggressive nature of SCLC can be attributed to various factors, including its rapid proliferation, frequent genetic mutations, and early metastasis to other organs. Unfortunately, SCLC is often diagnosed in advanced stages, and current treatment options are limited, resulting in poor prognosis and low survival rates [2, 3]. Despite an initial response to chemotherapy, SCLC frequently relapses and becomes resistant to further treatment [4]. Therefore, there is an urgent need to identify new molecular targets and develop effective therapies to improve the clinical outcomes for SCLC patients.

Tumor microenvironment (TME) is an emerging therapeutic target, especially for enhancing the efficacy of immunotherapies [5]. The interaction between cancer cells and other surrounding host cells, especially myeloid cells, present in TME, plays a critical role in tumor growth, metastasis, and the acquisition of drug resistance [6, 7]. T cells play key roles in the clearance of tumor cells; however, a majority of cases remain exhausted or inhibited in the TME [8, 9]. Immunotherapies in non-small cell lung cancer (NSCLC) have significantly improved patient survival [10]. However, the addition of immunotherapy to platinum-based frontline chemotherapy had a limited impact on survival in SCLC patients [10, 11]. Not much is known about the tumor and immune cell interactions in SCLC. SCLC TME harbors relatively fewer tumor-infiltrating lymphocytes than NSCLC [9, 12], which may explain why immune checkpoint-based immunotherapies are less effective in treating SCLC. This disparity in lymphocyte presence may explain the relatively lower effectiveness of immune checkpoint immunotherapies in SCLC.

S100A9 is a small calcium-binding protein and is increased during inflammation [13]. In addition, S100A9 expression is augmented in many solid tumors, promoting tumor growth, metastasis, drug resistance, and immune suppression [13–17]. S100A9 has been reported to act on tumor and immune cells [18]. S100A9 has been shown to modulate the myeloid-derived suppressor cell (MDSC) activity in the TME [19]. MDSCs have been shown to promote tumor progression by inhibiting T-cell functions and reducing the efficacy of chemotherapy and immunotherapies [20]. A specific chemical inhibitor of S100A9, Tasquinimod, has been used to treat solid tumors and has undergone phase III trials for recalcitrant prostate cancer [21, 22]. However, the role of S100A9 in regulating SCLC growth and metastasis and its downstream effects in modulating the functions of tumor and immune cells in TME have not been studied.

Our current study conducted a comprehensive analysis of S100A9 in the progression and spread of SCLC using various SCLC cell lines and patient samples. We also utilized different preclinical mouse models, including patient-derived xenograft (PDX) mouse models, to evaluate the effects of tasquinimod, a drug that inhibits S100A9 and can reduce the pro-tumor effects of S100A9. Our analysis of human SCLC patient samples showed that higher S100A9 expression in the tumor was linked to lower overall survival. Our findings indicate that higher S100A9 expression promotes SCLC growth and distant spread by reducing autophagy through MAGE-A3 in tumor cells. Furthermore, S100A9 increases the presence of MDSCs and decreases the recruitment of CD8+ T cells in the TME. This study identifies S100A9 signaling as a promising target for treating SCLC and demonstrates that pharmacological inhibition of S100A9 can improve the clinical outcome for SCLC patients with high S100A9 expression.

## Materials and methods

### Reagents and antibodies

RPMI (ATCC), DMEM (Corning), fetal bovine serum (FBS) (Sigma-Aldrich), penicillin and streptomycin (PS) antibiotic, and trypsin were obtained from Gibco. Tissue culture plastic wares were obtained from Corning. DAPI. Tasquinimod was purchased from MedChem Express (MCE). The EGFP-LC3 plasmid was obtained from Addgene.

### Cell culture

Human SCLC cell lines H69, H69-AR, H209, H146, and H82 were obtained from ATCC. SBC5 and SBC3 cell lines were a kind gift from Saburo Sone and Seiji Yano (University of Tokushima School of Medicine, Japan), and KP1 (murine) SCLC cells were received as a generous gift from Dr. Julien Sage (Stanford University). All SCLC cell lines were cultured in RPMI supplemented with 10% FBS and 1% penicillin/streptomycin at 37 °C and 5% CO_2_; however, SBC5 cells were cultured in DMEM supplemented with 10% FBS and 1% penicillin/streptomycin at 37 °C and 5% CO_2_. All cell lines were verified by STR profiling and routinely screened for mycoplasma contamination and confirmed to be free of mycoplasma.

### Tissue microarray

TMA slides containing paraffin-embedded SCLC patient tissues were processed at the Pathology Core Facility at Ohio State University. TMA includes a total of 60 SCLC samples with different grades and stages. Immunohistochemistry (IHC) on these slides was performed using the S100A9 antibody (Supplementary Table 1). All the slides were analyzed and scored by an expert pathologist in a blinded manner. S100A9 expression scores 0–1 were considered low, and 2–3 were considered high. A log-rank test was used for survival data analysis, and Kaplan–Meier survival curves were used to display the results. Holm’s procedure was used to adjust for multiple comparisons.

### ELISA and flow cytometry

Human and mouse S100A9 levels were measured by ELISA (R&D Systems) as per the manufacturer’s instructions. Single-cell suspensions from tumors were analyzed by flow cytometry as described earlier [23]. Briefly, a single cell suspension of tumor cells was blocked using an Fc blocker (BD Biosciences), followed by antibody staining for 30 min. After the antibody staining, cells were washed three times with PBS at 1500 rpm for 5 min at 4 °C and fixed with 4% paraformaldehyde, and data were recorded on a flow cytometer. Flow cytometry data analysis was performed using the FlowJo software. The primary antibodies used for flow cytometry are provided in (Supplementary Table 1).

### T cell proliferation assay

To assess T cell proliferation and activation in vitro, naive CD3+ T cells were freshly isolated using the kit and activated by plate-bound anti-mouse CD3 (mCD3) and anti-mouse CD28 (mCD28) antibodies with mouse IL-2 (mIL-2). These cells were stained with CellTrace Violet (CTV) for 20 min at 37 °C, and T cells were cultured in RPMI-1640 medium supplemented with 10% FBS, 2 mM L-glutamine, 100 units/mL penicillin, 100 μg/mL streptomycin, and 0.05 mM 2-mercaptoethanol at 37 °C/5% CO2. T cell proliferation was determined by CTV dilution, and cell viability was evaluated by 7AAD staining. Data was recorded on a flow cytometer. Flow cytometry data analysis was performed using the FlowJo software.

### Small interfering (si) RNA knockdown, migration, and wound healing assay

siRNAs targeting MAGE-A3, SNAIL, and RAGE were purchased from Dharmacon, and the transfection was performed as described earlier [24]. Migration and Wound healing assays were performed as described [25]. Briefly, cancer cells were grown in a serum-free medium on an 8-μm filter insert (Corning), and 10% FBS (200 μl) was used as a chemoattractant. Migrated cells were stained and counted on a bright-field microscope.

### Lentiviral production, Western blot (WB), and IHC

Specific oligonucleotides targeting S100A9 and a scramble control (Supplementary Table 1) were cloned into pLKO.1 cloning vector per the Addgene protocol (Cambridge, MA, USA). Lentiviral infections were performed as described earlier [26]. Briefly, lentivirus was produced in 293T cells, and cancer cells were transduced with the lentivirus with polybrene (8 µg/ml). Transduced cancer cells were selected using puromycin (2 µg/ml). WB and IHC were performed as described earlier [25].

### RNA sequencing and computational data analysis

RNA sequencing (RNA-seq) and data analysis were performed using the previously published sample preparation techniques and data analysis pipelines [27]. Briefly, total RNA was isolated from SBC5-scr or SBC5-S100A9-deficient cells using the RNeasy Plus Mini Kit (Qiagen). The quality of RNA was tested on a Bioanalyzer 2100 (Agilent), and RNA-seq was performed at the Ohio State University core facilities. Libraries were generated with NEBNext Ultra™ II Directional RNA Library Prep Kit for Illumina (NEB) and NEBNext Poly(A) mRNA Magnetic Isolation Module (NEB) with an input amount of 200 ng total RNA per sample. Libraries were pooled and sequenced on an Illumina NovaSeq SP platform (Illumina). Analysis performed with in-house pipeline BISR-RNAseq. Raw fastq was aligned to the human reference genome GRCh38 with hisat2 v2.1.0. Gene-wise counts were generated with feature Counts from the subread package v1.5.1 for genes annotated by Homo_sapiens. GRCh38.96, counting the primary alignment in the case of multi-mapped reads. Raw counts were normalized by voom, and differential expression was performed with limma. Genes were tested to see if at least 66% of the samples had an expression of 2 CPM. Three comparisons were made to assess differential expression between groups S100A9_scr vs. S100A9_sh1. Significant genes had FDR < 0.05 and logFC>1 or <-1. Heatmaps were generated using ComplexHeatmap. S100A9 gene expression data and overall survival data were obtained from the TCGA portal (www.cbioportal.org/public-portal/).

### Animal experiments

All animal experiments were approved by the Institutional Animal Care and Use Committee of the Ohio State University and performed as per our approved animal protocol (2007A0233-R5). Animals were housed with regard to food, water, and cages per University Laboratory Animal Resources guidelines. Animal experiments were performed using B6.129svF1 mice obtained from Jackson Laboratories and Nude mice obtained from targeted validation shared resources (TVSR) at the Ohio State University (OSU), USA. For subcutaneous tumor implantation, 5 × 10^5^ cells in 100 μl of Matrigel were injected into the right flank of mice. On the arrival of palpable tumors, mice were randomly divided into four groups and treated with tasquinimod (10 mg/kg) or cisplatin (2.5 mg/kg) until endpoints. Tumors were measured weekly using a digital caliper, and tumor volume was calculated using the following formula: width*width*length*0.52. For the patient-derived xenograft (PDX) experiment, we received the SCLC PDX (MSK_LX_298) as a kind gift from Dr. Charles M. Rudin (Memorial Sloan Kettering Cancer Center, New York, NY). The PDX was passaged and maintained in NSG mice. SCLC PDX was established by subcutaneous injection, and on the emergence of palpable tumors, mice were randomized and treated with vehicle control or tasquinimod (10 mg/kg) for four weeks.

### Statistical analysis

We performed the statistical analysis using GraphPad Prism 9 software (GraphPad Software Inc., San Diego, CA, USA). Data were expressed as mean ± SD. The means of all data were compared by unpaired *t*-test or one-way ANOVA. In each case, the *p*-value of <0.05 was considered statistically significant. Survival curves were plotted using the Kaplan–Meier method, and log-rank tests were used to compare curves between groups.

## Results

### S100A9 is aberrantly expressed and associated with poor overall survival in SCLC

**Fig. 1:**
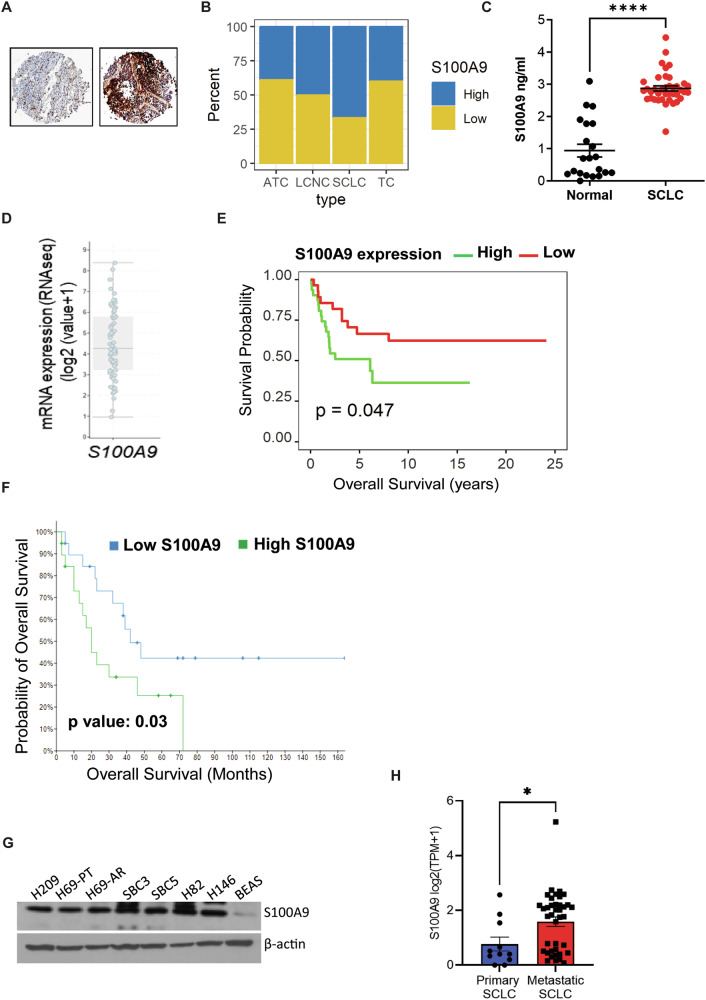
S100A9 is aberrantly expressed and associated with poor prognosis in SCLC. **A** Human lung cancer tissue microarray (TMA) was stained with S100A9 antibody using IHC. Representative images showing S100A9 expression levels in Atypical Carcinoid (ATC) (*n* = 25), Large Cell Neuroendocrine Carcinoma (LCNC) (*n* = 26), SCLC (*n* = 60), and Typical Carcinoid (TC) (*n* = 50). **B** The graph depicts the percentage of human lung cancer samples expressing different levels of S100A9. **C** S100A9 expression in normal (*n* = 21) and SCLC (*n* = 39) plasma samples was analyzed by ELISA. **D** The mRNA expression of S100A9 was obtained from the U Cologne study via cBioPortal. The mRNA expressions are depicted as Log2 (value + 1) from the RNA sequencing (RNA seq) dataset (*n* = 81 patient samples). **E** Using experimental TMA, the association of S100A9 protein levels with SCLC patient survival was analyzed. The Kaplan–Meier graph demonstrates the association between S100A9 expression and patient survival. **F** S100A9 expression was analyzed for correlation with the survival probability of SCLC patients using the data obtained from the U Cologne study via cBioPortal (*n* = 19). **G** Cell extracts prepared from SCLC cell lines were analyzed for S100A9 expression by Western blotting (β-actin was used as a loading control). **H** S100A9 mRNA expression was analyzed in primary and metastatic SCLC cell lines using the CCLE database. (*P < 0.05, ****P < 0.0001).

We found that S100A9 was highly expressed in lung cancer patients compared to other cancer types (Supplementary Fig. 1A). To explore the clinical significance of S100A9 in SCLC, we analyzed the protein expression of S100A9 in a lung cancer patient tissue microarray by immunohistochemistry (IHC). We found that S100A9 expression was significantly higher in SCLC samples (Fig. 1A, B). In addition, we observed that blood plasma levels of S100A9 were significantly higher in SCLC patient samples than in normal controls (Fig. 1C). Furthermore, we explored S100A9 expression in SCLC patients using cBioportal [28] and observed that S100A9 is highly expressed in SCLC patients (Fig. 1D).

Given high levels of S100A9 in SCLC patients, we investigated whether S100A9 expression had any impact on their overall survival using the S100A9 IHC protein expression data. We found that high S100A9 expression correlates with poor prognosis and poor overall survival in SCLC patients (Fig. 1E). Additionally, using cBioportal [28] data, we found that high S100A9 expression correlates with poor overall survival in SCLC patients (Fig. 1F).

We further analyzed an array of SCLC cell lines by Western blotting and found that S100A9 was overexpressed in SCLC cells compared to normal human bronchial epithelium (BEAS) cells (Fig. 1G). In addition, using ELISA, we observed that S100A9 expression was significantly higher in SCLC cells compared to BEAS cells (Supplementary Fig. 1B). Using the CCLE database, we found that S100A9 expression was enriched in metastatic SCLC cell lines compared to poorly metastatic SCLC cells (Fig. 1H). Our results suggest that S100A9 is overexpressed in SCLC and correlates with poor overall survival.

### S100A9 depletion reduces SCLC progression

**Fig. 2:**
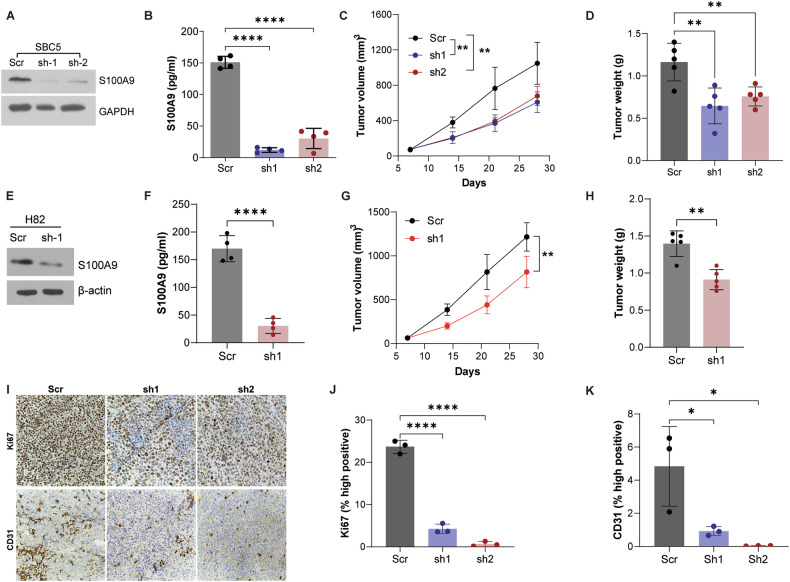
S100A9 depletion reduces tumor growth. The successful downregulation of S100A9 in SBC5 cells using an S100A9-specific short hairpin (sh) RNA was confirmed by (**A**) Western blotting (WB) and (**B**) ELISA. These cells were injected into nude mice, **C** tumor volume was measured weekly, and **D** tumor weight was analyzed at the end of the study. **E** S100A9 was knocked down in H82 cells, which was confirmed by WB and **F** ELISA. These cells were injected into nude mice. **G** Tumor volume was measured weekly, and **H** tumor weight was analyzed at the end of the study. **I** The expression of Ki-67 and CD31 was analyzed by IHC in tumors derived from scramble control (scr) and S100A9 knockdown (sh1 and sh2) tumors. **J**, **K** The percentage of high Ki-67 and CD31-positive cells were quantified. The data reported here is presented as mean ± SEM (*n* = 5). (*P < 0.05, **P < 0.01, ****P < 0.0001).

First, we evaluated the effect of S100A9 on SCLC colony formation and migration in vitro using S100A9 control and S100A9-depleted cells. We found that S100A9 depletion reduces colony-forming and migratory abilities of SBC5 and H82 SCLC cell lines in vitro (Supplementary Fig. 2A–D). Therefore, we further wanted to evaluate the impact of S100A9 depletion on SCLC growth in vivo. SBC5 cells were manipulated using two different shRNAs to stably downregulate S100A9 expression. S100A9 knockdown was confirmed by Western blotting and ELISA (Fig. 2A, B). These cells were used to establish SCLC tumors subcutaneously in the nude mice. We found that tumor development was markedly slower in S100A9 downregulated tumors compared to their respective controls (Fig. 2C, D).

In addition, we evaluated the oncogenic potential of S100A9 in another highly aggressive human SCLC cell line, H82. We used the S100A9-specific shRNA to knock down S100A9 expression (Fig. 2E, F). Similarly, we subcutaneously injected these modified cells into nude mice and monitored tumor growth. In agreement with the previous results, we observed a significant decrease in tumor growth upon S100A9 depletion compared to the scramble shRNA control group (Fig. 2G, H). Furthermore, decreased tumor cell proliferation (Ki-67 expression) and tumor neovascularization (CD31 expression) were observed in S100A9-downregulated tumors compared to controls (Fig. 2I–K). These findings strongly suggest a robust association between S100A9 expression and the rapid progression of SCLC.

### Tasquinimod is a promising therapeutic agent against SCLC progression and metastasis

**Fig. 3:**
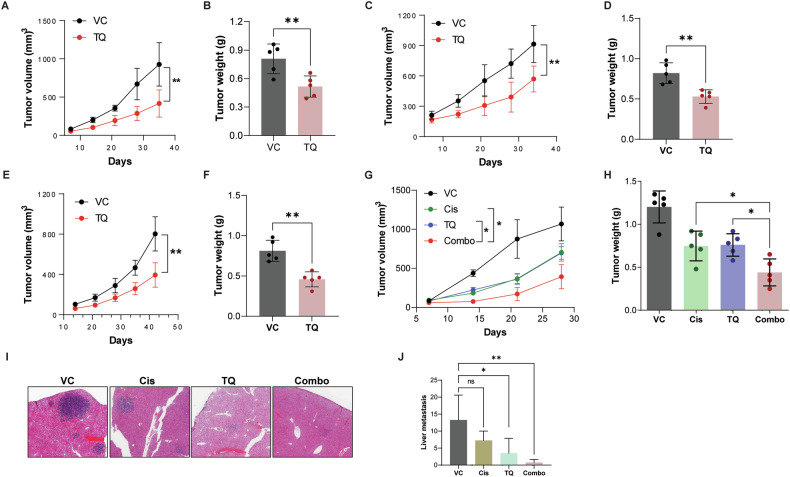
Tasquinimod inhibits tumor growth and metastasis in vivo. **A** SBC5 cells were implanted subcutaneously into nude mice (*n* = 5). Palpable tumors were treated with vehicle control (VC) or tasquinimod (TQ) for 4 weeks, and tumor volumes were measured externally every week during the tasquinimod treatment and **B** Tumor weight was calculated at the end of the experiment. Similarly, H82 cells were implanted into nude mice and treated with VC or TQ, and **C** tumor volume and **D** tumor weight were analyzed. **E**, **F** SCLC PDX was inoculated subcutaneously into the flanks of NSG mice. The PDX-bearing mice were treated with vehicle control (VC) or tasquinimod (TQ). Tumor volumes were measured externally every week, and tumor weight was calculated at the end of the experiment. **G**, **H** KP1 cells were implanted in B6.129svF1 mice, and the tumor-bearing mice were treated with vehicle control (VC) or tasquinimod (TQ) or cisplatin (Cis), or tasquinimod with cisplatin (combo). Tumor volume was measured weekly, and tumor weight was calculated at the end of the study. **I**, **J** Representative image of liver metastasis of KP1 tumors from different treatment groups and its quantification. The data reported here is presented as mean ± SEM (*n* = 5). (*P < 0.05, **P < 0.01, ns: non-significant).

Tasquinimod (ABR-215050; a quinoline-3-carboxyamide) is a small-molecule compound that targets S100A9 [21]. First, we assessed the effect of tasquinimod on the colony-forming and migratory abilities of SCLC cells in vitro. We observed that tasquinimod treatment led to a reduction in the colony-forming (Supplementary Fig. 3A-B) and migratory abilities of SBC5 and H82 cells (Supplementary Fig. 3C-D). Furthermore, the in vivo anti-tumor effect of tasquinimod treatment was assessed in various SCLC preclinical mouse models, like SBC5 and H82 SCLC xenografts, syngeneic murine SCLC, and SCLC PDX models. All these models have been shown to successfully generate xenograft tumors. Tasquinimod, selected for our in vivo studies, has a favorable safety profile and did not affect normal organ histology in animals. SBC5 xenograft models were treated with tasquinimod (10 mg/kg) over the course of 4 weeks, and the treatment significantly reduced the rate of tumor growth (Fig. 3A). In addition, the average tumor weight of tasquinimod-treated tumors was significantly lower compared to the control groups (Fig. 3B). Similarly, we tested the inhibitory effect of tasquinimod in H82 xenograft tumors. We found that tasquinimod treatment significantly reduced the tumor volume and weight compared to the control group (Fig. 3C, D).

A better understanding of human cancers in vivo can be gained by using the patient-derived xenograft (PDX) models. In the next step, we used an SCLC PDX (MSK_LX_298) model that showed high S100A9 protein expression by Western blotting (Supplementary Fig. 3E). In addition, we observed high MAGE-A3 expression in the SCLC PDX tumor (Supplementary Fig. 3F). We examined the inhibitory effect of tasquinimod in the SCLC PDX model. The results showed a significant inhibitory effect of tasquinimod on tumor growth in this PDX model (Fig. 3E, F). Since S100A9 inhibition alone using tasquinimod is not sufficient to eliminate tumors despite reduced tumor growth and metastasis in vivo, we next evaluated the ability of tasquinimod to sensitize tumors to cisplatin chemotherapy. Cisplatin is a well-established platinum-based chemotherapy agent in SCLC. We hypothesized that the combination of tasquinimod and cisplatin against SCLC is a promising therapeutic strategy due to their complementary mechanisms of action.

KP1 cells (murine SCLC cell line) show S100A9 expression (Supplementary Fig. 3G). Therefore, we used this syngeneic KP1 allograft model, which allowed us to interrogate the role of S100A9 signaling in an immune-intact environment. KP1 tumor-bearing B6129F1 immunocompetent mice were treated with tasquinimod (10 mg/kg) and/or cisplatin (2.5 mg/kg) over the course of 4 weeks. Mice treated with cisplatin or tasquinimod alone showed an adequate anti-tumor response; however, the tumor regression was much more pronounced in the tasquinimod and cisplatin combination treatment group (Fig. 3G, H). Notably, we did not observe any significant body weight loss with the combination treatment in these mice (Supplementary Fig. 3H). Furthermore, we observed reduced liver metastasis in tasquinimod-treated animals compared to the vehicle control treatment group (Fig. 3I, J). These results suggest that S100A9 is crucial for fueling SCLC growth, and its inhibition reduces the progression of the disease.

### Tasquinimod reduces the accumulation of MDSCs and increases activated CD8+ T cell recruitment in tumors

**Fig. 4:**
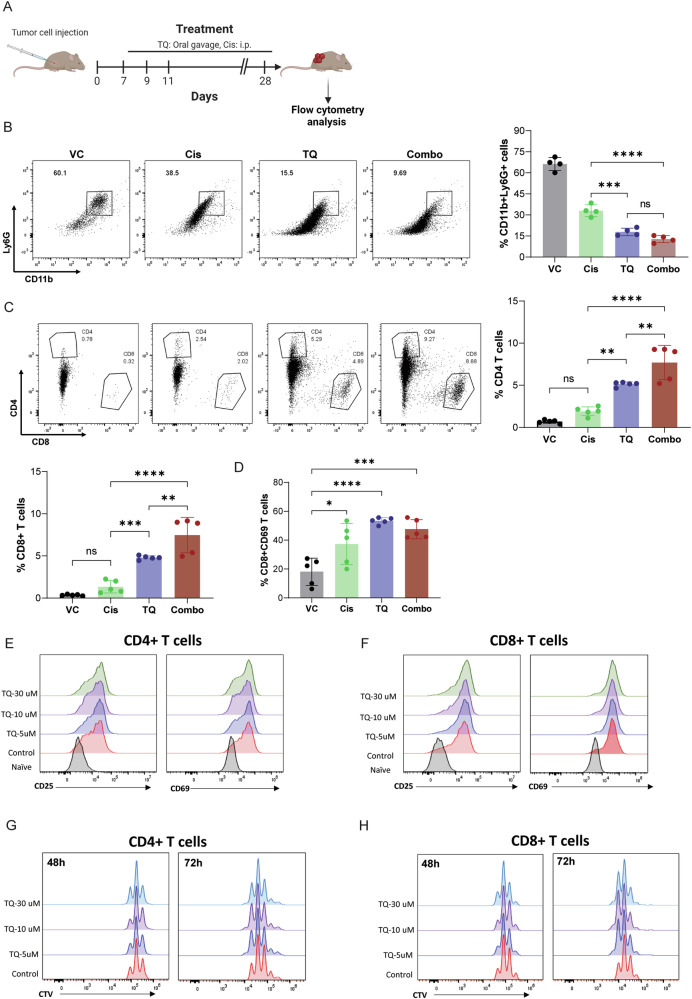
Tasquinimod treatment promotes tumor immunity. **A** The experimental schema used to test the effects of tasquinimod (TQ) and cisplatin (Cis) alone or in combination (i.p. intraperitoneal) on KP1 tumor growth. **B** KP1 tumors treated with vehicle control (VC) or TQ or Cis, or TQ + Cis combination (combo) were analyzed for the recruitment of MDSCs by flow cytometry. **C** KP1 tumors were assessed for the infiltration of CD4+ and CD8+ T cells by flow cytometry. **D** Tumor-infiltrated CD8+ T cells were analyzed for the expression of CD69 by flow cytometry. **E**, **F** Naive CD3+ T cells were activated by plate-bound anti-CD3 and anti-CD28 antibodies, and the activation of CD4+ and CD8+ T cells was analyzed by measuring CD25 and CD69 levels by flow cytometry at the indicated time points. **G**, **H** Cell proliferation was determined using CTV staining by flow cytometry. (*P < 0.05, **P < 0.01, ***P < 0.001, ****P < 0.0001, ns: non-significant).

To better understand the mechanisms underlying the observed combinatorial antitumor effect of tasquinimod and cisplatin, we profiled different subsets of immune cells infiltrating the tumor. Tumors were analyzed by multispectral flow cytometry to examine the changes in immune cells and tumor-infiltrating lymphocytes (TILs). TME comprises different types of immune cells, including a heterogeneous population of MDSCs that are known to promote tumor growth and metastasis by enhancing the immune-suppressive TME [29]. S100A9 is an important chemoattractant for MDSCs homing to tumors from the general circulation [30]. Tasquinimod treatment alone or combined with cisplatin significantly reduced the recruitment of granulocytic MDSCs (Cd11b+/Ly6G+) to the tumor compared to vehicle control or cisplatin alone treatment groups (Fig. 4B). In addition, tasquinimod-treated SBC5 or H82 tumors showed reduced MDSC accumulation (Supplementary Fig. 4A-B). Furthermore, we observed that tumors derived from S100A9-downregulated SBC5 cells showed reduced MDSC enrichment compared to SBC5 scramble (scr) cells (Supplementary Fig. 4C).

We profiled different subsets of T cells infiltrating the tumor to better understand the mechanisms underlying the observed combinatorial antitumor effect of tasquinimod and cisplatin. Treatment of tasquinimod alone or in combination with cisplatin significantly increased CD4+ and CD8+ T cell infiltration into the tumor compared to vehicle control or cisplatin alone treatment groups (Fig. 4C). Additionally, activated CD69+ CD8+ T cell subsets were significantly enriched in groups treated with tasquinimod alone and in combination with cisplatin compared to vehicle control (Fig. 4D).

Next, we analyzed the effect of S100A9 on T cell proliferation using the recombinant mouse S100A9 protein. We observed no significant changes in T cell proliferation (Supplementary Fig. 4D). We also assessed the direct impact of different doses of tasquinimod, ranging from 5 µM to 30 µM, on T cell activation and proliferation. Our findings indicated that tasquinimod treatment does not significantly influence the activation markers (CD25 and CD69) or the proliferation (as measured by CTV staining) of CD4+ and CD8+ T cells (Fig. 4E–H). Thus, tasquinimod treatment substantially enhances the anti-tumor immune responses by reducing the abundance of immunosuppressive MDSCs and promoting T-cell infiltration and activation in SCLC.

### S100A9 promotes SCLC cell survival through MAGE-A3 and Akt/GSK3α/β regulation

**Fig. 5:**
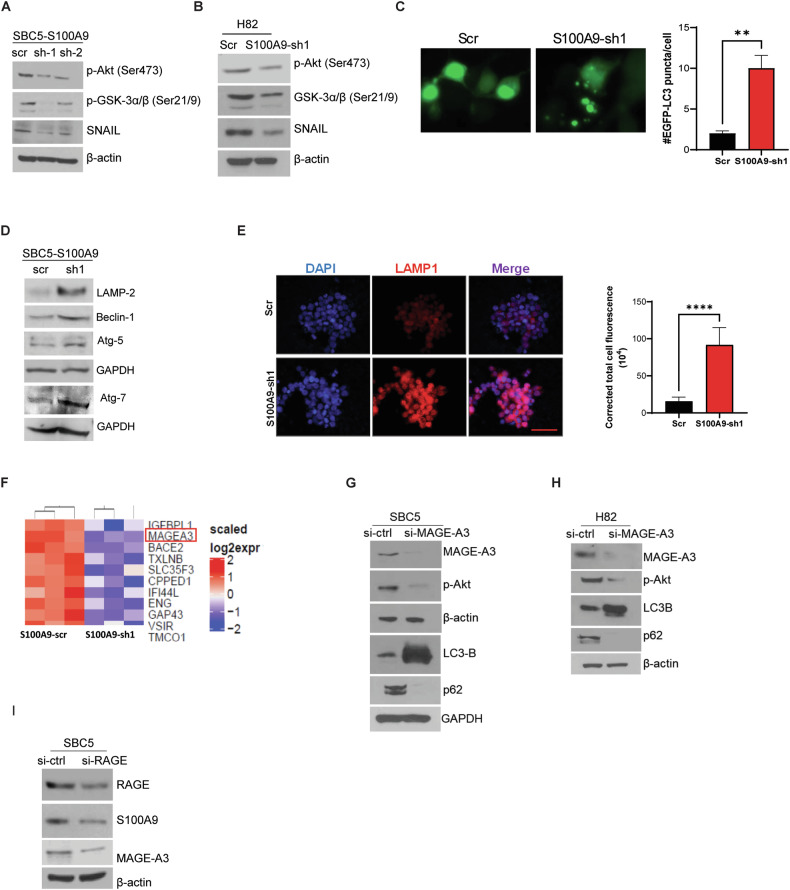
S100A9 enhances survival and reduces autophagy through MAGE-A3 regulation. **A** Phosphorylation and expression status of cell survival proteins in S100A9-scr and S100A9-sh1 SBC5 cells were analyzed by WB, and similarly, **B** H82 S100A9-scr and S100A9-sh1 cells were analyzed. **C** SBC5 cells were transfected with a plasmid containing GFP-LC3, and the cells were analyzed for LC3 puncta formation (quantified on right). **D** S100A9-scr and S100A9-sh1 SBC5 cells were analyzed for the expression of various autophagy-related proteins by WB. **E** S100A9-scr and S100A9-sh1 SBC5 cells were analyzed for the expression of LAMP1 using an immunofluorescence assay (quantified on the right). **F** Scramble control (S100A9-scr) and S100A9 downregulated (S100A9-sh1) SBC5 were analyzed by RNA-seq. MAGE-A3 expression (indicated by the red box). **G** MAGE-A3 was knocked down in SBC5 cells using a MAGE-A3-specific siRNA, and the changes in activation and expression of Akt and LC3-B, and p62 were analyzed by WB. Similarly, **H** MAGE-A3 knockdown-associated changes were analyzed in H82 cells. **I** RAGE was knocked down in SBC5 cells, and the expression of S100A9 and MAGE-A3 was analyzed by WB. The data reported here is the mean ± SEM of triplicate experiments. (**P < 0.01, ****P < 0.0001).

We observed that S100A9 plays an important role in SCLC survival. Therefore, we utilized a human phospho-proteome array to analyze the effect of S100A9 on cell survival pathways. We observed that S100A9 knockdown reduces the activation of Akt (Ser473, active form) and inhibitory phosphorylation of GSK3α/β (Ser-21/9, inactive form) (Supplementary Fig. 5A**)**. Akt is reported as a key cell survival signaling protein [31, 32]. We further confirmed our results in two different SCLC cell lines (SBC5 and H82) by analyzing the cell lysates from scramble control and S100A9-depleted SCLC cells by Western blotting. We found reduced phosphorylation of Akt (Ser-473) (Fig. 5A, B). Furthermore, we observed decreased inhibitory phosphorylation of GSK3α/β (Ser-21/9) (Fig. 5A, B). GSK3α/β is a critical downstream target of the Akt pathway that regulates the expression of various signaling molecules, including β-catenin and Snail [32, 33]. While we did not observe any changes in β-catenin activation (Supplementary Fig. 5B), we observed lower levels of Snail with S100A9 depletion (Fig. 5A, B). Snail is another important transcription factor known to promote EMT [32, 34]. In addition, higher levels of Snail have been reported to increase cancer cell migration [35]. We performed a transwell migration assay using Snail downregulated SCLC cells and observed a significantly reduced migratory potential of Snail-depleted SCLC cells compared to scramble controls (Supplementary Fig. 5C).

We further evaluated the effect of tasquinimod on the regulation of the Akt/GSK3α/β/Snail signaling pathway in SBC5 and H82 cells. Consistent with our previous results, we observed decreased activation of Akt and reduced inhibitory phosphorylation of GSK3α/β (Supplementary Fig. 5D-E). Additionally, tasquinimod treatment led to a reduction in Snail expression (Supplementary Fig. 5D-E). These results strongly suggest that S100A9 regulates Akt/GSK3α/β/Snail signaling axis to drive cell survival and migration in SCLC cells.

Interestingly, we observed that S100A9 depletion caused an increase in tumor cell autophagy, a cellular process associated with cancer cell death [36]. We observed significantly higher LC3 puncta formation, a marker of autophagy-induced cell death [37] in S100A9 downregulated cells than scramble controls (Fig. 5C). Furthermore, we found that S100A9 depletion caused an increase in autophagy-related proteins such as LAMP2, LAMP1, Beclin1, Atg7, and Atg5 (Fig. 5D, E). Next, to gain mechanistic insights, we performed RNA sequencing on S100A9 downregulated cells and observed a significant downregulation of melanoma-associated antigen A3 (MAGE-A3) (Fig. 5F). We further confirmed MAGE-A3 downregulation in S100A9-downregulated SBC5 and H82 cells by Western blotting (Supplementary Fig. 5F). Additionally, we found a positive correlation between S100A9 and MAGE-A3 expression in an SCLC patient dataset analysis [38] (Supplementary Fig. 5G). MAGE-A3 is a cancer-testis antigen, which is normally expressed in male germ cells, but it can also be expressed in other types of tumors, including lung cancer and melanoma [39]. However, the underlying tumor-promoting mechanisms of MAGE-A3 remain poorly understood. Recently, MAGE-A3 has been shown to inhibit tumor cell autophagy to enhance cell survival [40]. We interrogated the role of MAGE-A3 in inhibiting autophagy by knocking down MAGE-A3 expression in SCLC cells (SBC5, H82). MAGE-A3 knockdown caused an increase in autophagy-associated protein, LC3B, and a decrease in p62, which negatively correlates with autophagy (Fig. 5G, H). In addition, we observed a significant increase in LC3 puncta formation following MAGE-A3 knockdown (Supplementary Fig. 5H). Furthermore, we observed decreased Akt phosphorylation (Ser-473) (Fig. 5G, H). S100A9 has been reported to mediate its effect through the RAGE receptor; therefore, we also used a RAGE-specific siRNA for RAGE knockdown in SBC5 cells. We observed the reversal of S100A9-mediated signaling effects, such as reduced S100A9 and MAGE-A3 expression (Fig. 5I). Mechanistically, our results suggest that tumor cell-secreted S100A9 binds to RAGE in an autocrine manner and regulates the activation of Akt/GSK3α/β/Snail for increasing cell survival and migration.

### S100A9 inhibition conferred a survival advantage in SCLC

**Fig. 6:**
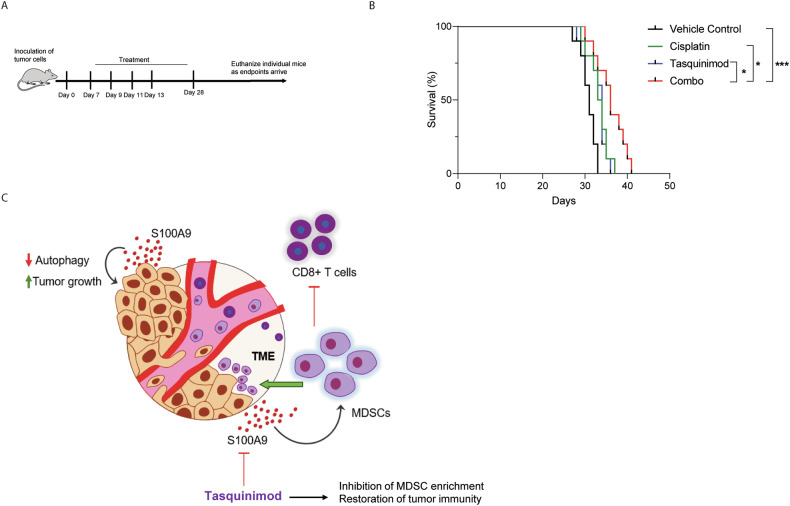
Targeting S100A9 increases overall survival in SCLC. **A** The experimental schema used to test the effects of tasquinimod with or without cisplatin in the KP1 subcutaneous SCLC model. **B** After the emergence of palpable tumors, mice were randomized for treatments until the study endpoints were reached. Survival curves were generated by the Kaplan–Meier method. **C** Schematic of S100A9-mediated tumor-promoting and immunosuppressive effects in the SCLC tumor microenvironment. (*P < 0.05, ***P < 0.001).

Based on the oncogenic roles of S100A9 in SCLC, we postulated that S100A9 inhibition in combination with cisplatin chemotherapy might provide added survival benefits for SCLC patients. Using the KP1 subcutaneous tumor model, we observed that S100A9 inhibition alone or in combination with cisplatin demonstrated a significant survival advantage over cisplatin or tasquinimod administration alone (Fig. 6A, B). Altogether, these promising preclinical results underscore the potential of targeting S100A9 in combination with standard-of-care chemotherapy to significantly improve the clinical efficacy of cisplatin-based chemotherapy and extend the overall survival of SCLC patients.

## Discussion

The results presented in this study provide significant insights into the clinical significance and functional role of S100A9 in SCLC. The findings demonstrate that S100A9 is aberrantly expressed in SCLC patients and is associated with poor overall survival. This observation is supported by analyzing patient tissue samples and publicly available datasets, which consistently show high S100A9 expression in SCLC. Furthermore, the in vivo studies using SCLC xenograft and syngeneic murine models demonstrate that downregulating S100A9 significantly reduces tumor growth, accompanied by reduced expression of proliferative and angiogenic markers. The study also investigates the therapeutic potential of tasquinimod, a promising agent against SCLC. We have shown that tasquinimod treatment exhibits a significant anti-tumor effect in xenograft models, patient-derived xenograft models, and a syngeneic murine model. In addition, we have demonstrated that tasquinimod treatment combined with cisplatin chemotherapy against SCLC resulted in an improved survival rate. Tasquinimod has been shown to be effective and well-tolerated in castrate-resistant prostate cancer trials (NCT01732549, NCT01234311). Since tasquinimod targets the immunosuppressive microenvironment, combining it with other immunotherapies could be a promising approach for treating SCLC patients and warrants further investigation.

The study also provides important insights into the SCLC immune microenvironment and its modulation by S100A9 inhibition. Immunosuppressive mechanisms are reported to promote tumorigenesis and help in immune escape [41]. In lung tumors, infiltrated myeloid cells create an immunosuppressive microenvironment for enhanced tumor growth [42]. Immunosuppressive granulocytes in SCLC patients are associated with poor clinical outcomes and pose a significant therapeutic challenge [43]. Tasquinimod treatment, either alone or in combination with cisplatin, reduces the accumulation of MDSCs, which are well known for their plastic nature and are often exploited by cancer cells to create an immunosuppressive TME [44]. Tumor-secreted soluble factors act as a chemoattractant for accumulating MDSCs in the TME to support the growth of primary tumors [45]. These suppressive MDSC cells are significantly high in SCLC and decrease the clinical efficacy of immunotherapies [46]. No known effective treatments for targeting these MDSCs in the TME exist. Nonetheless, our results demonstrate that inhibiting S100A9 in tumor cells either by genetic means or using an S100A9 inhibitor significantly impedes the recruitment of immunosuppressive granulocytic MDSCs in the tumor. Notably, these MDSCs in tumors have been reported to suppress the proliferation and effector functions of tumor-reactive CD8+ T cells [47]. We showed that tasquinimod treatment increases the infiltration of activated CD8+ T cells in the tumor. Our study suggests that S100A9 suppresses the anti-tumor immune responses by enhancing the enrichment of granulocytic MDSCs and reducing the recruitment and activation of cytotoxic T cells within SCLC tumors (Fig. 6C).

Furthermore, mechanistic insights into the S100A9-mediated signaling revealed that S100A9 mediates its tumor-intrinsic effects by binding to the RAGE receptor in an autocrine loop. S100A9 orchestrates cell survival and migration by regulating the Akt/GSK3α/β/Snail signaling axis. We discovered that in tumor cells, autocrine S100A9/RAGE signaling regulates the expression of MAGE-A3, inhibiting autophagy and increasing cell survival. MAGE-A3 is reported to regulate tumor cell autophagy [40, 48]. Autophagy plays a complex role in cancer biology. Based on our findings, the data indicate that S100A9 offers pro-survival benefits by suppressing autophagy in SCLC cells. In addition, autophagy-mediated mechanisms facilitate the processing and presentation of tumor antigens to T cells [49]. This antigen presentation enhances tumor-specific immune responses [50]. Autophagy induction through S100A9 inhibition may also contribute to enhanced CD8+ T cell infiltration and activation in SCLC. Further investigation is needed to identify how these proteins regulate autophagy and their implications in SCLC progression.

In summary, the results presented in this study offer compelling evidence for the clinical importance and functional role of S100A9 in SCLC. The findings endorse the potential of targeting S100A9 as a therapeutic approach and underscore the effectiveness of tasquinimod in inhibiting SCLC progression, modulating the immune tumor microenvironment, and enhancing overall survival. These insights enhance our understanding of SCLC pathogenesis and may steer the development of new therapeutic strategies for this aggressive subtype of lung cancer.

## Supplementary information

Supplementary Figure 1

Supplementary Figure 2

Supplementary Figure 3

Supplementary Figure 4

Supplementary Figure 5

Full and uncropped western blot

Supplementary Figure Legends

Supplementary Table

## Data Availability

All data supporting the findings of this study are included in the main text or the supplementary materials.
