# Supplementary figures and images for: S100A9 enhances tumor immune suppression and cancer cell survival in small cell lung cancer
Source: Cell Death Dis. 2025 Oct 31;16(1):774. doi: 10.1038/s41419-025-08102-0 (PMC12578924; doi:10.1038/s41419-025-08102-0)

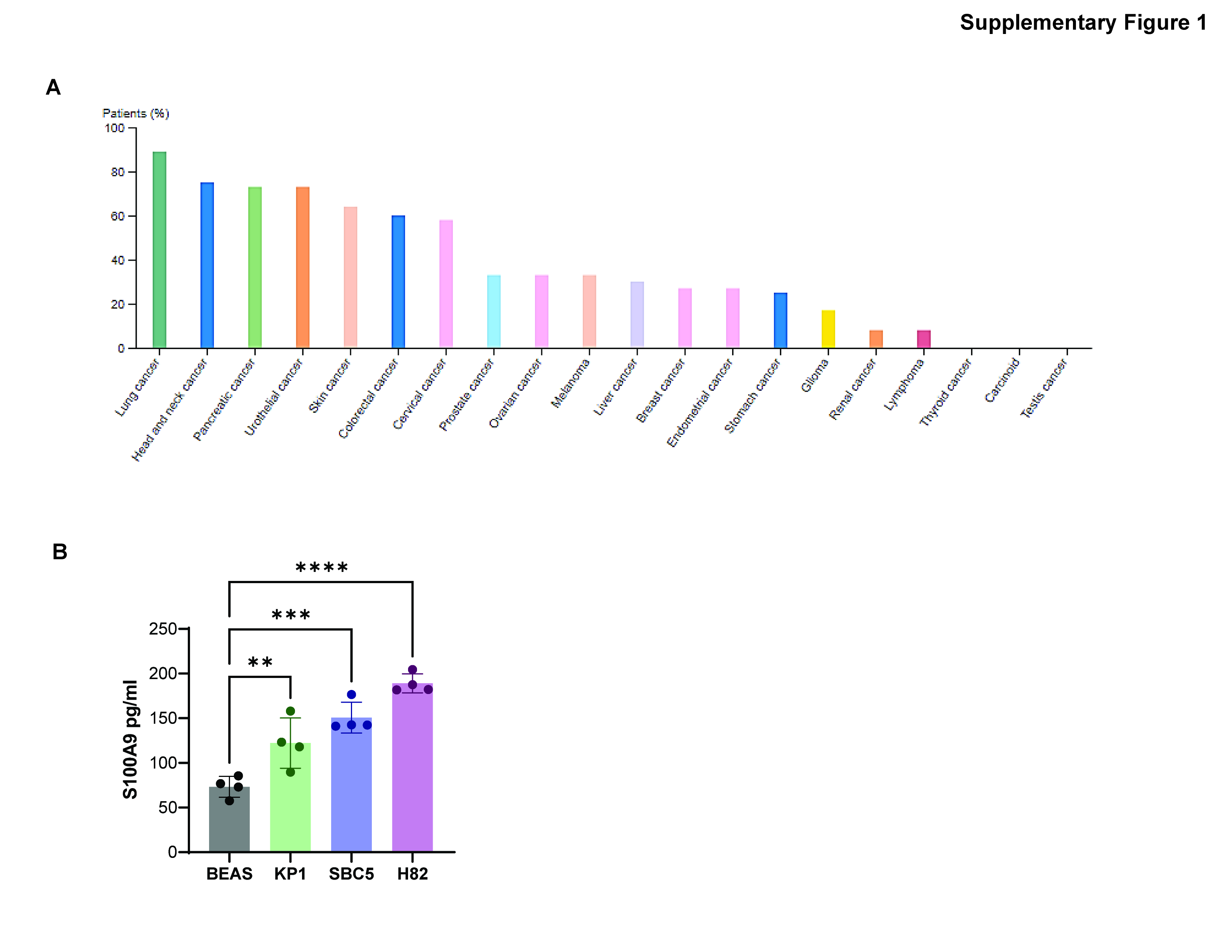

Supplement: Supplementary file 1 — Supplementary Figure 1 [file 41419_2025_8102_MOESM1_ESM.tif]

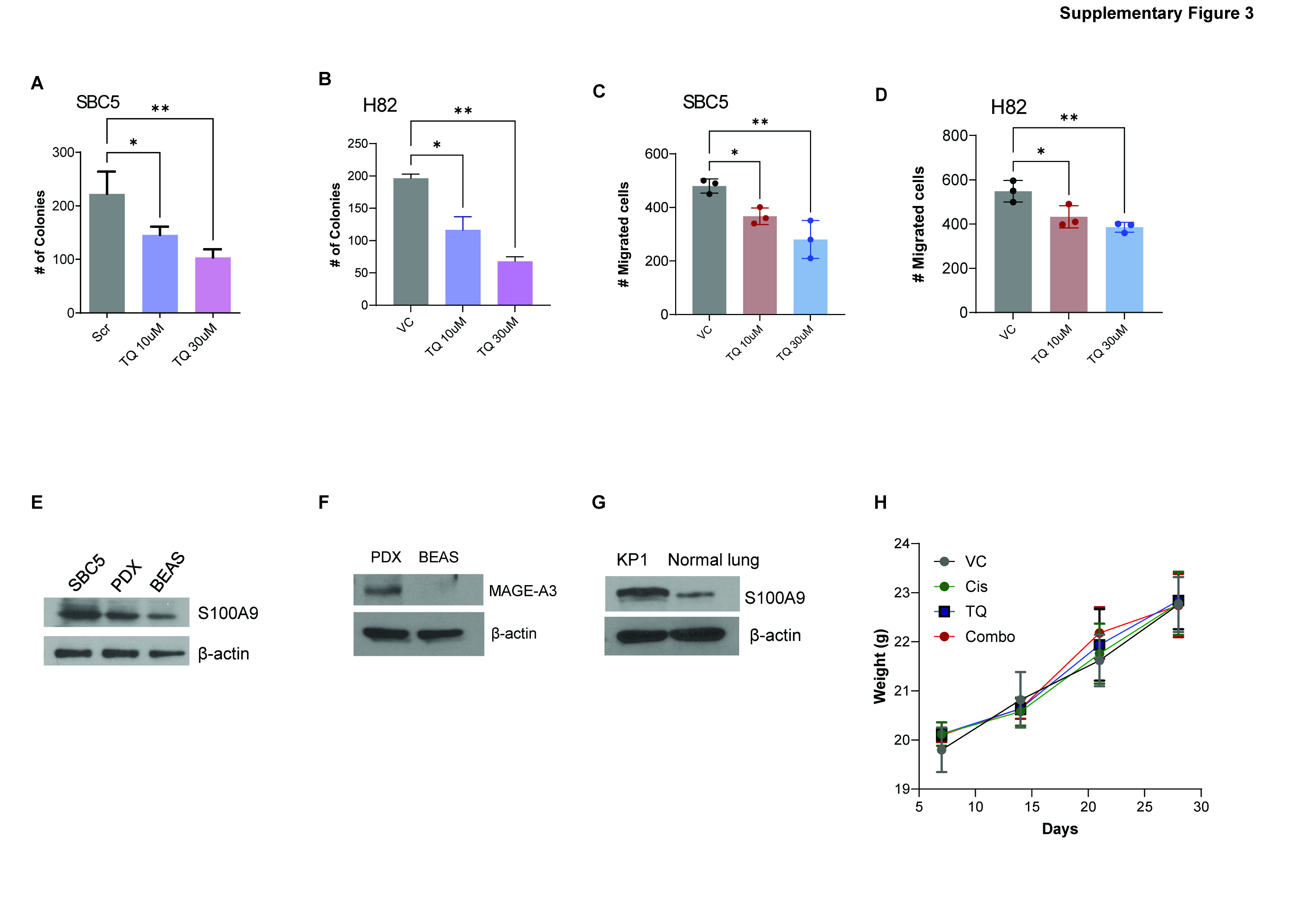

Supplement: Supplementary file 3 — Supplementary Figure 3 [file 41419_2025_8102_MOESM3_ESM.tif]

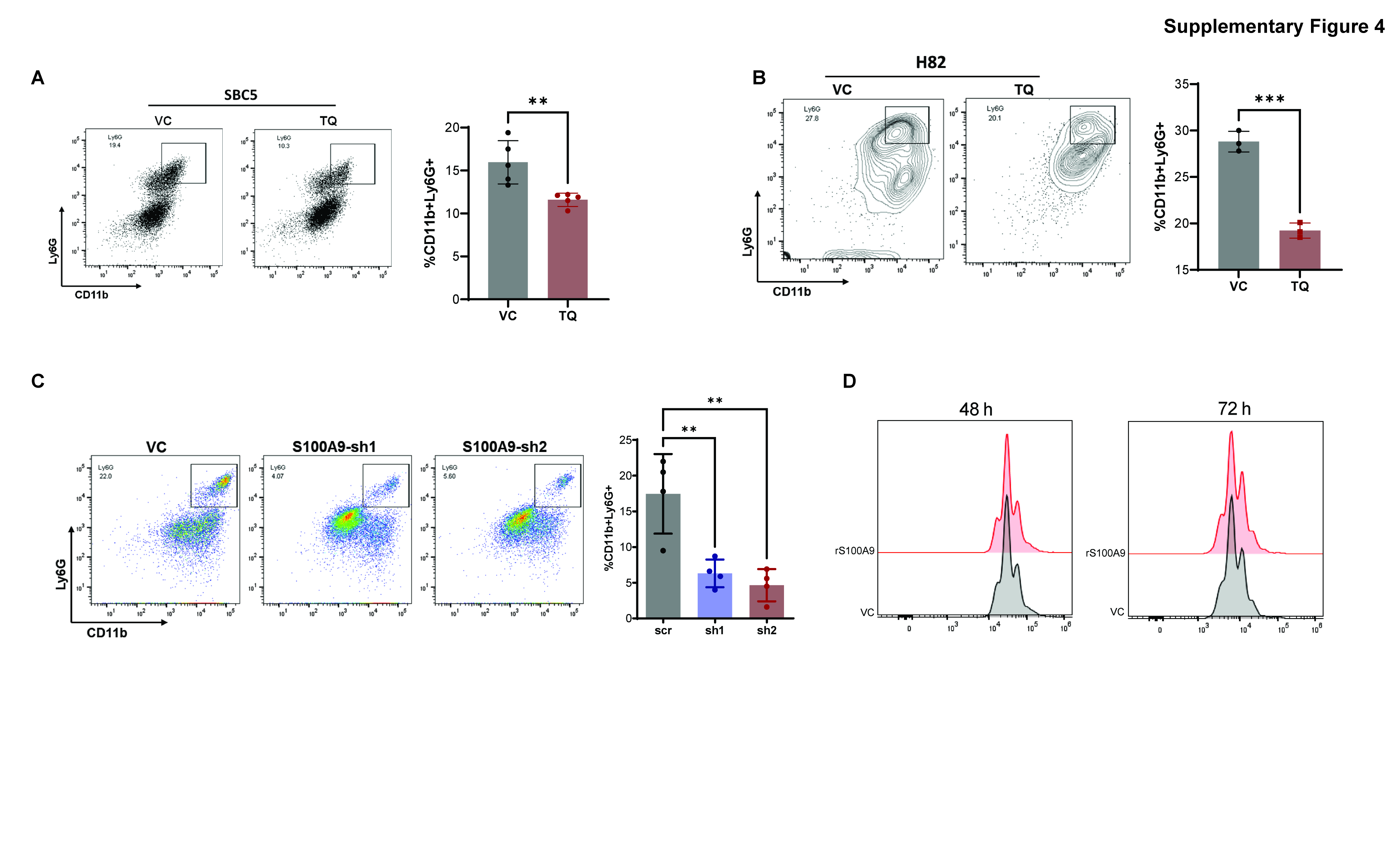

Supplement: Supplementary file 4 — Supplementary Figure 4 [file 41419_2025_8102_MOESM4_ESM.tif]

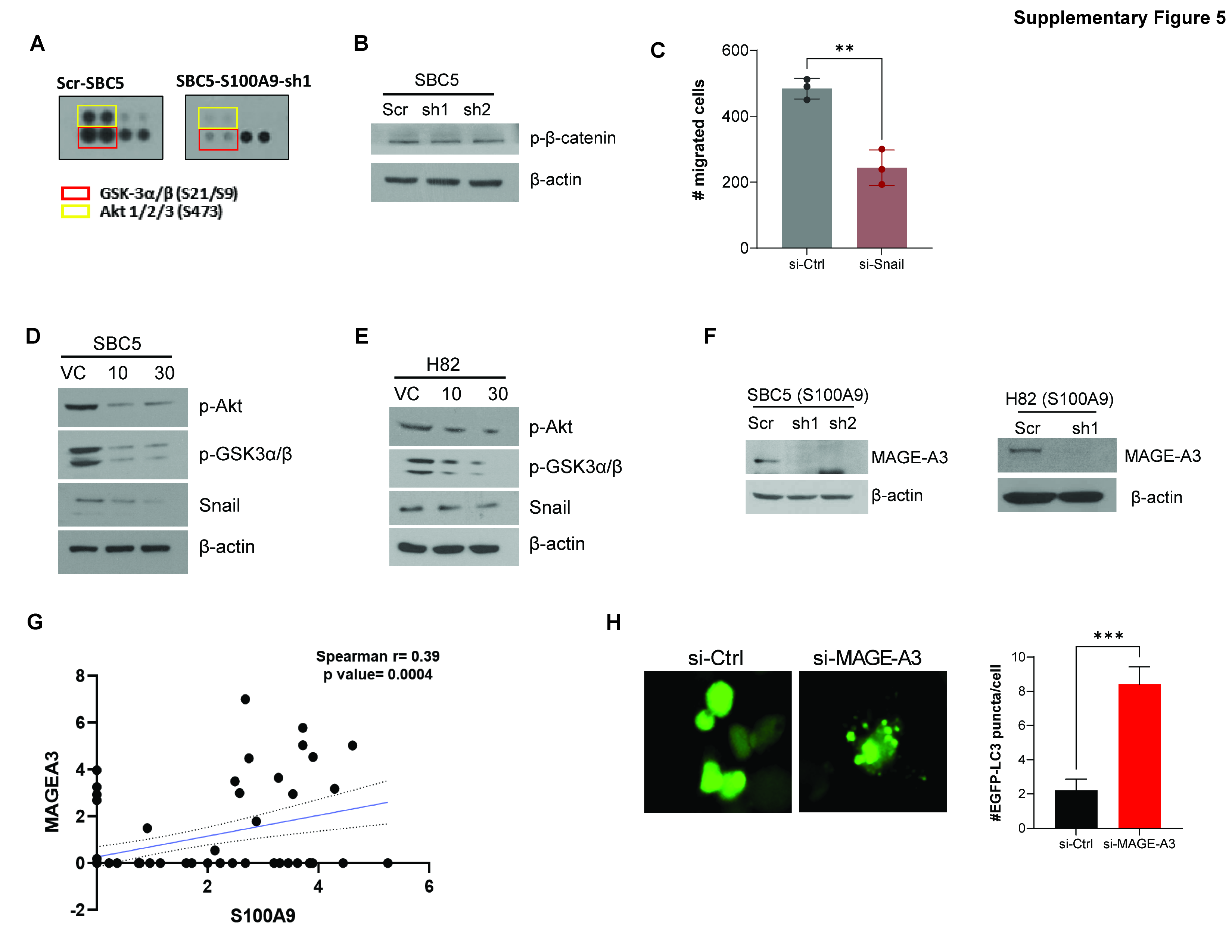

Supplement: Supplementary file 5 — Supplementary Figure 5 [file 41419_2025_8102_MOESM5_ESM.tif]
