# Supplementary material for: S100A9 enhances tumor immune suppression and cancer cell survival in small cell lung cancer
Source: Cell Death Dis. 2025 Oct 31;16(1):774. doi: 10.1038/s41419-025-08102-0 (PMC12578924; doi:10.1038/s41419-025-08102-0)
Supplement: Supplementary file 7 — Supplementary Figure Legends [file 41419_2025_8102_MOESM7_ESM.docx]

**Supplementary Figure Legends:**

**Supplementary Figure 1:** S100A9 is highly expressed in lung cancer. (A) We analyzed the Human Protein Atlas ([www.proteinatlas.org](http://www.proteinatlas.org)) database and found that S100A9 was highly expressed in lung cancer. (B) We analyzed the conditioned medium obtained from different SCLC cell lines and estimated S100A9 expression by ELISA.

**Supplementary Figure 2:** S100A9 depletion reduces colony-forming and migratory abilities of SCLC cells. Scramble control or S100A9 downregulated SBC5 and H82 cells were seeded in low numbers and allowed to grow for 10-15 days, followed by cell staining. Colonies were quantified. These cells were also analyzed for migratory abilities by transwell assay.

**Supplementary Figure 3:** (A-B) Tasquinimod treatment inhibits colony-forming abilities of SCLC cells. (C-D) Tasquinimod treatment reduces the migratory abilities of SCLC cells. (E) S100A9 expression was analyzed in SCLC PDX (MSK_LX_298) by Western blotting. (F) MAGE-A3 expression was analyzed in SCLC PDX (MSK_LX_298) by Western blotting. (G) S100A9 expression was examined in the KP1 cell line by Western blotting. (H) KP1 tumor-bearing mice were weighed once a week during the course of treatment.

**Supplementary Figure 4:** S100A9 inhibition/downregulation reduces MDSC enrichment in tumors. (A-B) Single-cell suspension of tasquinimod or vehicle control-treated SBC5 or H82 tumors were analyzed for the MDSC recruitment by flow cytometry. (C) Single-cell suspension of tumors derived from scramble control or S100A9-downregulated SBC5 cells were analyzed for the MDSC recruitment by flow cytometry. (D) Naive CD3⁺ T cells were activated with plate-bound anti-CD3 and anti-CD28 antibodies and treated with recombinant mouse S100A9 (100 ng/mL). CD8⁺ T cell proliferation was assessed by CTV staining and analyzed via flow cytometry.

**Supplementary Figure 5:** S100A9 enhances migration and survival in SCLC cells. (A) scramble control or S100A9 downregulated SBC5 cells were analyzed for the downstream changes in the activation and expression of various proteins by a human phospho-proteome array. (B) p-β-catenin phosphorylation was analyzed in scramble control and S100A9-downregulated SBC5 cells by Western blotting. (C) Control or Snail siRNA-treated SBC5 cells were analyzed for migration using a transwell migration assay. (D) SBC5 and (E) H82 cells were treated with vehicle control and different doses of tasquinimod, and the changes in the activation and expression of Akt, GSK3α/β, and snail were analyzed by Western blotting. (F) MAGE-A3 expression was analyzed in S100A9-downregulated SBC5 and H82 cells by Western blotting. (G) The correlation between S100A9 and MAGE-A3 expression was analyzed using an SCLC patient dataset (GEO ID: GSE60052). (H) Control and MAGE-A3-downregulated SBC5 cells were transfected with a plasmid containing GFP-LC3, and the cells were analyzed for LC3 puncta formation (quantified on right).

**Original Data (Supplementary Figure):** Uncropped Western blots.
