# Supplementary material for: S100A9 enhances tumor immune suppression and cancer cell survival in small cell lung cancer
Source: Cell Death Dis. 2025 Oct 31;16(1):774. doi: 10.1038/s41419-025-08102-0 (PMC12578924; doi:10.1038/s41419-025-08102-0)
Supplement: Supplementary file 8 — Supplementary Table [file 41419_2025_8102_MOESM8_ESM.docx]

**Supplementary Table 1**

**1**

| **Antibodies** | **Dilution** | **Source** | **Identifier** |
| --- | --- | --- | --- |
| S100A9 | 1:1000 | Abcam | ab92507 |
| p-Akt (Ser473) | 1:1000 | CST | 4060S |
| t-Akt | 1:1000 | CST | 4691S |
| p-GSK3α/β (Ser-21/9) | 1:1000 | CST | 9327S |
| MAGE-A3 | 1:1000 | CST | 38896S |
| p62 | 1:1000 | CST | 5114S |
| LC3B | 1:1000 | CST | 3868S |
| LAMP2 | 1:1000 | CST | 49067S |
| Beclin1 | 1:1000 | CST | 3495S |
| Atg7 | 1:1000 | CST | 8558S |
| GAPDH | 1:5000 | CST | 92310SF |
| β-actin | 1:10000 | CST | 3700S |
| Atg5 | 1:1000 | CST | 9980S |
| Live/Dead Zombie aqua | 1:1000 | Biolegend | 423101 |
| CD45 | 1:400 | Biolegend | 103133 |
| CD3 | 1:200 | Biolegend | 100219 |
| CD4 | 1:200 | BD Biosciences | 612952 |
| CD8 | 1:200 | BD Biosciences | 612759 |
| CD69 | 1:100 | Biolegend | 104543 |
| CD11b | 1:200 | Biolegend | 101215 |
| Ly6G | 1:200 | Biolegend | 127607 |
| Recombinant Murine IL-2 | 5 ng/ml | Peprotech |  |
| Anti-mouse CD3 | 2-5 μg/ml | BioXcell, | BE0001 |
| Anti-mouse CD28 | 2-5 μg/ml | BioXcell | BE0015 |
| CellTrace Violet (CTV) | 5µM | Invitrogen | C34557 |

**s**hRNA primer sequences:

| **Primer name** | **Forward (5’-3’)** | **Reverse (5’-3’)** |
| --- | --- | --- |
| Scramble (scr) | CCGGCCTAAGGTTAAGTCGCCCTCGCTCGAGCGAGGGCGACTTAACCTTAGGTTTTTG | AATTCAAAAACCTAAGGTTAAGTCGCCCTCGCTCGAGCGAGGGCGACTTAACCTTAGG |
| S100A9-sh1 | CCGGAAGGTCATAGAACACATCATGCTCGAGCATGATGTGTTCTATGACCTTTTTTTG | AATTCAAAAAAAGGTCATAGAACACATCATGCTCGAGCATGATGTGTTCTATGACCTT |
| S100A9-sh2 | CCGGCACCAATACTCTGTGAAGCTGCTCGAGCAGCTTCACAGAGTATTGGTGTTTTTG | AATTCAAAAACACCAATACTCTGTGAAGCTGCTCGAGCAGCTTCACAGAGTATTGGTG |
